# Dehydrocorydaline Protects Against Sepsis-Induced Myocardial Injury Through Modulating the TRAF6/NF-κB Pathway

**DOI:** 10.3389/fphar.2021.709604

**Published:** 2021-08-19

**Authors:** Yadong Li, Li Zhang, Ping Zhang, Zhiying Hao

**Affiliations:** ^1^ Department of Emergency, Second Hospital of Shanxi Medical University, Taiyuan, China; ^2^ Department of Hemotology, Renmin Hospital of Wuhan University, Wuhan, China; ^3^ Department of Pharmacy, Shanxi Cancer Hospital, Taiyuan, China

**Keywords:** sepsis, myocardial injury, dehydrocorydaline, TRAF6/NF-κB pathway, inflammation

## Abstract

We aim to investigate the effect and mechanism of dehydrocorydaline (Deh), an alkaloidal component isolated from *Rhizoma corydalis*, in the treatment of sepsis-mediated myocardial injury. Lipopolysaccharide (LPS) was taken to construct an *in-vitro* sepsis-myocardial injury models H9C2 cardiomyocytes. The *in-vivo* model of sepsis in C57BL/6 mice was induced by intraperitoneal injection of *Escherichia coli* (*E. coli*). The *in-vitro* and *in-vivo* models were treated with Deh in different concentrations, respectively. Hematoxylin-eosin (HE) staining, Masson staining, and immunohistochemistry (IHC) staining were taken to evaluate the histopathological changes of the heart. ELISA was applied to evaluate the levels of inflammatory factors, including IL-6, IL-1β, TNFα, IFNγ, and oxidized factors SOD, GSH-PX in the plasma or culture medium. Western blot was used to measure the expressions of Bax, Bcl2, Caspase3, iNOS, Nrf2, HO-1, TRAF6, NF-κB in heart tissues and cells. The viability of H9C2 cardiomyocytes was detected by the CCK8 method and BrdU assay. The ROS level in the H9C2 cardiomyocytes were determined using immunofluorescence. As a result, Deh treatment improved the survival of sepsis mice, reduced TUNEL-labeled apoptosis of cardiomyocytes. *In vitro,* Deh enhanced the viability of LPS-induced H9C2 cardiomyocytes and inhibited cell apoptosis. Additionally, Deh showed significant anti-inflammatory and anti-oxidative stress functions *via* decreasing IL-1β, IL-6, TNFα, and IFNγ levels, mitigating ROS level, up-regulating Nrf2/HO-1, SOD, and GSH-PX expressions dose-dependently. Mechanistically, Deh inhibited TRAF6 expression and the phosphorylation of NF-κB p65. The intervention with a specific inhibitor of TRAF6 (C25-140) or NF-κB inhibitor (BAY 11-7082) markedly repressed the protective effects mediated by Deh. In conclusion, Deh restrains sepsis-induced cardiomyocyte injury by inhibiting the TRAF6/NF-κB pathway.

## Introduction

Sepsis is a clinical syndrome that mainly manifests as a systemic inflammatory response caused by infection, and has a high mortality rate in countries around the world (Innocenti et al., 2020). Septic myocardial injury, as one of the most prevailing clinical complications, is commonly observed in the late stage of severe sepsis, and the mortality rate of patients with sepsis combined with cardiac dysfunction is 70–90% (Hochstadt et al., 2011). The crucial factors of myocardial damage are changes in hemodynamics, inflammation, mitochondrial damage and oxidative stress induced by sepsis (Lewis et al., 2012). On the other hand, unbalanced oxidative stress following sepsis also contributes to the damage of mitochondrion functions, which leads to the insufficient production of mitochondrial ATP, resulting in fatal energy failure of the cardiomyocytes, and ultimately leads to myocardial injury (Rocha et al., 2012). Therefore, studying the mechanism of sepsis-induced inflammation and oxidative stress helps the treatment of sepsis-induced myocardial injury.

TLR4, one member of toll-like receptors (TLRs), has been found to exert a prominent effect on sepsis occurrence and maintenance (Oyama et al., 2004; Hennessy et al., 2010). Lipopolysaccharide (LPS) is a major component of the gram-negative bacilli cell wall. As a pathogen-related molecular pattern, it can be specifically recognized by TLR4 to initiate intracellular signal transduction, thereby mediating immune and inflammatory responses (Zeuke et al., 2002; Ha et al., 2008). Previous studies on myocardial injury have demonstrated that a number of drugs alleviate LPS-mediated myocardial injury by regulating TLR4. For example, Clemastine Fumarate contributes to the prevention of myocardial ischemia-reperfusion injury by regulating the TLR4/PI3K/Akt signaling pathway (Yuan et al., 2020). Furthermore, ginsenoside Rg1 alleviates myocardial cell apoptosis and inflammation by inhibiting the TLR4/NF-κB/NLR family pyrin domain containing three signaling pathway (Luo et al., 2020).

TNF receptor-associated factor 6 (TRAF6) was one of the first identified downstream signal proteins of TLRs. Located at the central point of the convergence of signals induced by TLR and TNF receptor families, TRAF6 is widely involved in the inflammatory response and immune response mainly by mediating inflammation and apoptotic signaling pathways (Inoue et al., 2007). TRAF6 has an important effect on the activation of the intracellular signal transduction pathway mediated by TLR4. For instance, the limitation of bromodomain and extraterminal domain protein expression inhibits the TLR4/TRAF6/NF-κB signaling pathway, thus restraining acute myocardial infarction (Sun et al., 2015). Therefore, examining the function and mechanism of TLR4/TRAF6 in LPS-induced myocardial injury is expected to reveal a novel therapeutic approach for sepsis-induced myocardial injury.

2,3,9,10-tetramethoxy-13-methyl-5,6-dihydroisoquinolino [2,1-b] isoquinolin-7-ium (Deh; molecular formula, C_22_H_24_NO_4_) is a quaternary ammonium salt alkaloid extracted from fumarine (Huo et al., 2018). Deh has been reported to have beneficial effects in the treatment of depression and melanoma. For instance, Deh can alter the content of monoamine in brains by limiting uptake-2 monoamine transporters, thus exerting its antidepressant effects (Jin et al., 2019). Additionally, it restrains cell proliferation, migration and invasion by inhibiting the MEK1/2-ERK1/2 signaling pathway in melanoma (Hu et al., 2019). Furthermore, Deh limits the increase of mitochondrial membrane potential and ATP depletion in macrophages induced by LPS and inhibits the release of IL-1β and IL-6 through macrophages (Ishiguro et al., 2011). Therefore, exploring the effects of Deh on sepsis-mediated myocardial injury may be of interest.

The present study revealed that Deh enhanced the viability of cardiomyocytes in an *Escherichia coli* (*E. coli*)- or LPS-induced sepsis model, inhibited cell apoptosis, reduced IL-1β and TNFα levels, and upregulated the expressions of superoxide dismutase (SOD) and glutathione peroxidase (GSH-PX) in the plasma and culture medium. Additional experiments demonstrated that Deh alleviated LPS-induced myocardial injury *via* the inhibiting TRAF6/NF-κB signaling pathway and activating Nrf2/HO-1 pathway. Collectively, the present study revealed the protective mechanism of Deh in myocardial injury, providing a reference for sepsis-mediated myocardial injury treatment.

## Materials and Methods

### Sepsis Animal Model and Drug Treatment

A total of 80 male C57B6/L mice (age, 8–10 weeks, 20–22 g) were obtained from and kept in the Animal Research Center of the Shanxi Medical University (animal license: SYXK (Jin)2021-0001). All mice were kept under controlled lighting (light: dark, 12:12 h) and temperature (22 ± 1°C) conditions with free access to food and water for 10 days to acclimate to the environment. The mice were randomly divided into six groups, including sham group (*n* = 10 per group), Deh group (20 mg/kg, *n* = 10), Sepsis group (*n* = 15), Sepsis + Deh (5 mg/kg group, *n* = 15), Sepsis + Deh (10 mg/kg group, *n* = 15), Sepsis + Deh (20 mg/kg group, *n* = 15). Deh was administered *via* intraperitoneal injection (once every 24 h). An *in vivo* sepsis-induced myocardial injury model was constructed as previously described (Song et al., 2018). Briefly, a total number of 3.5 × 10^6^ *Escherichia coli* (ATCC25922) was injected intraperitoneally into the mice. After 30 min, the mice in the Sepsis group were treated with Deh (5, 10 and 20 mg/kg body weight) *via* intraperitoneal injection for three consecutive days. Deh (cat. no. N2090; purity >98%) was obtained from APeXBIO Technology LLC and the treatment dose was selected based on a previous study (Bin et al., 2019). The survival rate of mice in each group was monitored hourly. 6 h or 24 h after the *Escherichia coli* injection, the peritoneal lavage fluid and serum were collected for further analysis.

### Liver and Kidney Function Test

Following centrifugation (500 g for 10 min at 4°C), the supernatant of peritoneal lavage fluid and serum was collected, stored at −80°C and used to examine the levels of plasmatic inflammatory factors (IFNγ,IL-6, IL-1β and TNFα) and antioxidant factors (SOD and GSH-PX) using their detection kits. For the evaluation of liver and kidney function, the levels of creatinine, urea nitrogen, aspartate aminotransferase (AST), and alanine aminotransferase (ALT) in the supernatant of peritoneal lavage fluid and serum were measured using an automatic biochemical analyzer (AU800; Olympus Corporation, Tokyo, Japan).

### Bacterial Counts

The collected peritoneal lavage fluid and serum were subjected to serial log fold dilution in sterile saline. 10-fold serial dilutions of the bacterial load in blood and peritoneal lavage fluid were plated on tryptic soy blood agar plates, which were incubated for 24 h at 37 C. After the incubation, the bacterial colonies were counted and expressed as log CFU/ml blood or peritoneal fluid.

### Heart Tissue Collection

All mice received deep anesthesia by intravenous injection of 100 mg/kg phenobarbital sodium (CAS:57-30-7, Sigma-Aldrich). The hearts of the mice were removed, and one half of the hearts was used for histopathological evaluation and the other half was used for western blotting. All procedures were conducted following the Guidelines for the Care and Use of Laboratory Animals issued by the National Institutes of Health (NIH publication, 2011 revision). All animal studies have been approved by the Ethics Committee of the Shanxi Cancer Hospital (Taiyuan, China, Approval number: SXZL-2021-LL-008).

### Cardiomyocyte Culture and Treatment

H9C2 cells (Cat.No. CRL-1446, American Type Culture Collectionm, USA), derived from rat embryonic ventricular myocytes, were cultured in high glucose (4,500 mg/l) DMEM (Gibco; Thermo Fisher Scientific, Inc.) supplemented with 10% FBS (Gibco; Thermo Fisher Scientific, Inc.), 100 U/ml penicillin (Beijing Solarbio Science and Technology Co., Ltd.) and 100 mg/ml streptomycin (Wuhan Fortuna Chemical Co., Ltd.). Cells were maintained in a standard culture dish (Becton, Dickinson and Company) at 37°C with 5% CO_2_ in a wet environment on a monolith, and the medium was replaced every 2 days.

### Cell Grouping and Treatment

H9C2 cells were divided into the following groups: 1) Control; 2) Deh (20 μg/ml); 3) LPS; 4) LPS + Deh (5 μg/ml); 5) LPS + Deh (10 μg/ml); 6) LPS + Deh (20 μg/ml); 7) LPS + C25-140; 8) LPS + C25-140 + Deh (10 μg/ml); 9) LPS + BAY 11-7082; 10) LPS + BAY 11-7082 + Deh (10 μg/ml). Inflammatory damage was induced by 10 μg/ml LPS treatment. The cells were dealt with *E. coli* LPS (10 μg/ml; serotype 0111: B4; Sigma-Aldrich; Merck KGaA) to induce an *in-vitro* sepsis model. The TRAF6 inhibitor C25-140 (Cat. No.HY-120934, MedChemExpress), and the NF-κB inhibitor BAY 11-7082 (Cat. No. HY-13453, MedChemExpress), were used for pretreatment of the cells at a concentration of 5 and 1 µM for 2 h, respectively.

### Cell Counting Kit 8 Assay

The viability of H9C2 cells was examined using a CCK8 (Beyotime Institute of Biotechnology) assay according to the manufacturer’s protocols. Briefly, H9C2 cells were inoculated into 96-well plates (1 × 10^5^ cells/ml) and incubated for 24 h in a 37°C incubator with 95% O_2_ and 5% CO_2_. After the treatments were finished, 10 μl CCK8 reagent was added to each well. Subsequently, the cells underwent a 3-h incubation at 37°C. Ultimately, the absorbance value was determined at 450 nm. Each experiment was repeated three times and each measurement was made three times.

### ELISA

After the treatment was finished, the culture medium of H9C2 cells was collected and the cells in the medium were removed by centrifugation at room temperature. The levels of the inflammatory factors (IFNγ,IL-6, IL-1β and TNFα) and oxidative stress factors (SOD and GSH-PX) in the mouse plasma supernatant or cell culture medium were determined, and the experiments were performed strictly according to the requirements of the kit. The aforementioned detection kits, including IFNγ (Cat.No. 70-EK280/3-96), IL-6 (Cat.No. 70-EK206/3-96), IL-lβ (Cat.No. 70-EK201B/3-96) and TNFα (Cat.No. 70-EK282/3-96) were purchased from MultiSciences (Hangzhou, China). The detection kits of SOD (Cat. No. A001-3-2) and GSH-PX (Cat. No. A005-1-2) were purchased from Nanjing Jiancheng Bioengineering Institute (Nanjing, China).

### Western Blotting

Following treatment of mouse myocardial tissues or H9C2 cells, the heart tissues and cells were collected. Subsequently, RIPA lysis buffer (Roche Diagnostics) was used for isolation of total protein. The total nucleoprotein was extracted using a Nucleoprotein Extraction Kit [Order NO. C500009, Sangon Biotech (Shanghai) Co., Ltd.]. Total protein (50 μg) was subjected to SDS-PAGE at 100 V for ∼2 h. Next, the separated proteins were electrically transferred to the PVDF membranes. Then, the membranes were blocked with 5% skimmed milk powder for 1 h at room temperature, rinsed with TBS with 0.1% Tween-20 (TBST) three times for 10 min each, and incubated overnight at 4°C with TRAF6 (dilution, 1:1,000; cat. no. ab218575; Abcam), phosphorylated-(p-) NF-κB (phospho S536) (dilution, 1:1,000; cat. no. ab86299; Abcam), NF-κB (dilution, 1:1,000; cat. no. ab32536; Abcam), iNOS (dilution, 1:1,000; cat. no. ab178945; Abcam), Nrf2 (dilution, 1:1,000; cat. no. ab62352; Abcam), Heme Oxygenase 1 (dilution, 1:1,000; cat. no. ab68477; Abcam), Histone H3 (dilution, 1:1,000; cat. no. ab1791; Abcam), Bax (dilution, 1:1,000; cat. no. ab32503; Abcam), Bcl2 (dilution, 1:1,000; cat. no. ab182858; Abcam), and Caspase3 (dilution, 1:1,000; cat. no. ab13847; Abcam) primary antibodies. After washing with TBST, the membranes were incubated with HRP-labeled anti-rabbit or anti-mouse secondary antibodies (dilution, 1:300, cat. no. ab205718; Abcam) for 1 h at room temperature. Subsequently, TBST was used to wash the membranes three times for 10 min each. Finally, western blot reagent (Invitrogen; Thermo Fisher Scientific, Inc.) was added for color imaging. ImageJ (Version: 1.52v, National Institutes of Health) was used to analyze the gray values of each protein.

### 5-Bromo-2-Deoxyuridine Assay

A single cell suspension was prepared from H9C2 cells in each group during the logarithmic growth period and cells were inoculated into 24-well plates at a density of 1 × 10^5^ cells/well until the cells grew adherent to the wall. BrdU labeling reagent (Sigma-Aldrich; Merck KGaA) was added according to the manufacturer’s protocols, and the plates were placed in an incubator with 5% CO_2_ at 37°C. Following continuous cell culture for 48 h, the cells underwent immunofluorescence staining according to the manufacturer’s protocols of the BrdU Cell Proliferation Assay kit (Sigma, Shanghai, China). The nucleus was stained by DAPI (Beyotime, Shanghai, China). The positive cell quantity and the total number of DAPI-positive cells in three randomly selected fields were calculated and observed under a microscope. Cell proliferation rate = number of BrdU-positive cells/number of DAPI-positive cells. The average cell proliferation rate of three visual fields was taken as the cell proliferation rate.

### H&E Staining

Myocardial tissues of mice were washed with normal saline and fixed with 4% paraformaldehyde for 30–50 min. Subsequently, the samples were washed, dehydrated, cleaned, soaked in wax, embedded and sliced. The sections were heated in an incubator at 45°C, dewaxed, rinsed with alcohol of high to low concentration and distilled water for 5 min, stained with hematoxylin for 5 min, rinsed 3 s with tap water, rinsed with 1% hydrochloric acid ethanol for 3 s, and stained with 5% eosin for 3 min. Following dehydration, the sections were cleaned, installed and observed under a microscope.

### Immunohistochemical Staining

Sections (5-μm thick) of the paraffin-embedded heart tissues were dewaxed in water, heated in pH 6.0 antigen repair solution three times for 5 min each and washed with PBS. Subsequently, 3% hydrogen peroxide solution was added. Sections were incubated in the dark for 20 min at room temperature, blocked with 5% BSA (Beyotime, Shanghai, China) for 30 min at room temperature, washed and incubated overnight at 4°C with TRAF6 (dilution, 1:500; cat. no. ab40675; Abcam) antibody. Then, the sections were washed two times with PBS and incubated for 50 min at room temperature. Following three washes with PBS, the sections were incubated for 4 min with 3,3′ diaminobenzidine tetrahydrochloride, and the positive color was tan brown. Next, the sections were rinsed with running water until the color faded. The sections were counterstained with hematoxylin for ∼3 min, washed with tap water, underwent 1% hydrochloric acid ethanol differentiation for several seconds, and were washed with tap water until the ammonia turned blue. Subsequently, the sections were rinsed with tap water again. Following dehydration and blocking, the membranes were placed under a microscope for examination, image acquisition and analysis, and the integrated optical density was calculated using Image-Pro Plus (Version: 6.0, National Institutes of Health) software. For the detection of apoptotic cells in the heart, the TUNEL Assay Kit-HRP-DAB (cat. no. ab66110; Abcam) was used and the experimental processes were performed according to the instructions of the manufacturer. The nucleus was stained by DAPI (Beyotime, Shanghai, China). The number of TUNEL positive cells (every 0.1 mm^2^) was counted using Image-Pro Plus (Version: 6.0, National Institutes of Health) software.

### Masson Staining

The hearts of mice were isolated, fixed with 4% paraformaldehyde, embedded by paraffin and sectioned. the Masson’s trichrome staining was performed using Masson’s Trichrome Stain Kit (Cat.No.G1340, Solarbio, Beijing, China) according to the instructions of the producer. The sections were treated sequentially with hematoxylin and ferric oxide, acid fuchsin, phosphomolybdic acid, and acetic acid, and then neutral gum was used for the mounting of the sections.

### RT-PCR

RT-PCR was used to detect TRAF6 mRNA level both in the heart and H9C2 cells. TRIzol reagent (Invitrogen, USA) was used to isolate total RNA from the tissues or cells according to the manufacturer’s instructions. Next, the RNA was reverse-transcribed into cDNA using the Superscript First Strand Synthesis System (Invitrogen, USA). Then the qPCR amplification was analyzed using real-time quantitative PCR (SYBR Green) with an ABI-7900 Sequence Detection System (Applied Biosystems, USA). The relative expression of TRAF6 mRNA was quantified by the 2^−ΔΔCt^ method using GAPDH as the internal control. The primers used in this study include: TRAF1, forward primers, 5′-CGA​GGT​TGG​CAT​GAC​TTG​AG-3′, reverse primers, 5′-GCT​CAA​TGT​CCA​TGC​CTC​AG-3’; TRAF2, forward primers, 5′- CTT​CTC​CCC​AGC​CTT​CTT​CA-3′, reverse primers, 5′-AAT​GCG​TCG​ATC​ACA​TGC​TC-3′; TRAF3, forward primers, 5′- AGG​TGT​CCT​CCC​TGC​TAG​TA -3′, reverse primers, 5′- ACA​CTA​CAG​GAA​GCT​GGG​AC -3′; TRAF4, forward primers, 5′- CTG​GAG​GAG​CTA​TCT​GTG​GG-3′, reverse primers, 5′- CGA​GGA​GAT​TGT​CAA​AGG​CG-3′; TRAF5, forward primers, 5′- TGA​GGT​CTT​TGG​TGG​ATG​CT-3′, reverse primers, 5′- CCA​AAT​GAG​CTT​GCC​ACT​GT-3′; TRAF6, forward primers, 5′-GGA​AGA​GCA​GTC​GTT​TCC​TG-3′, reverse primers, 5′-GTC​ACA​CCT​CTA​CGG​GGA​AA-3′; TRAF7, forward primers, 5′- CCT​GTC​TGG​TGT​CTC​TGT​GT-3′, reverse primers, 5′- ACA​GTT​TGC​ACC​CCT​GGA​TA-3′; GAPDH, 5′-ACC​ACA​GTC​CAT​GCC​ATC​AC-3′, reverse primers, 5′-TCC​ACC​ACC​CTG​TTG​CTG​TA-3′.

### Reactive Oxygen Sepsis Detection

For evaluating the oxidative stress level in H9C2 cells, the DCFDA/H2DCFDA - Cellular ROS Assay Kit (ab113581, Abcam) was used. H9C2 cells were plated into a 6-well plate at a density of 3 × 105/well. The cells were incubated in a 5% CO2 incubator at 37°C. After the treatment on H9C2 cells was finished, they were washed with PBS, stained with 2′,7′-dichlorofluorescin diacetate (DCFDA) solution (20 µM) for 45 min at 37°C. Next, the cells were washed with 1 × Buffer for three times. The fluorescence signal was detected and observed using Olympus microscopy (BX53, Japan).

### Statistical Analysis

SPSS software (version 20.0; IBM Corp.) was used for data analysis, and all data are presented as the mean ± SD. Correlation was analyzed using a Pearson’s correlation test. Student’s *t*-test was performed for statistical analysis of two-group data, while the difference between the two groups was analyzed using a χ^2^ test. The survival of mice was analyzed using the Kaplan Meier method. *p* < 0.05 was considered to indicate a statistically significant difference.

## Results

### Deh Attenuates Sepsis-Mediated Myocardial Injury

**FIGURE 1:**
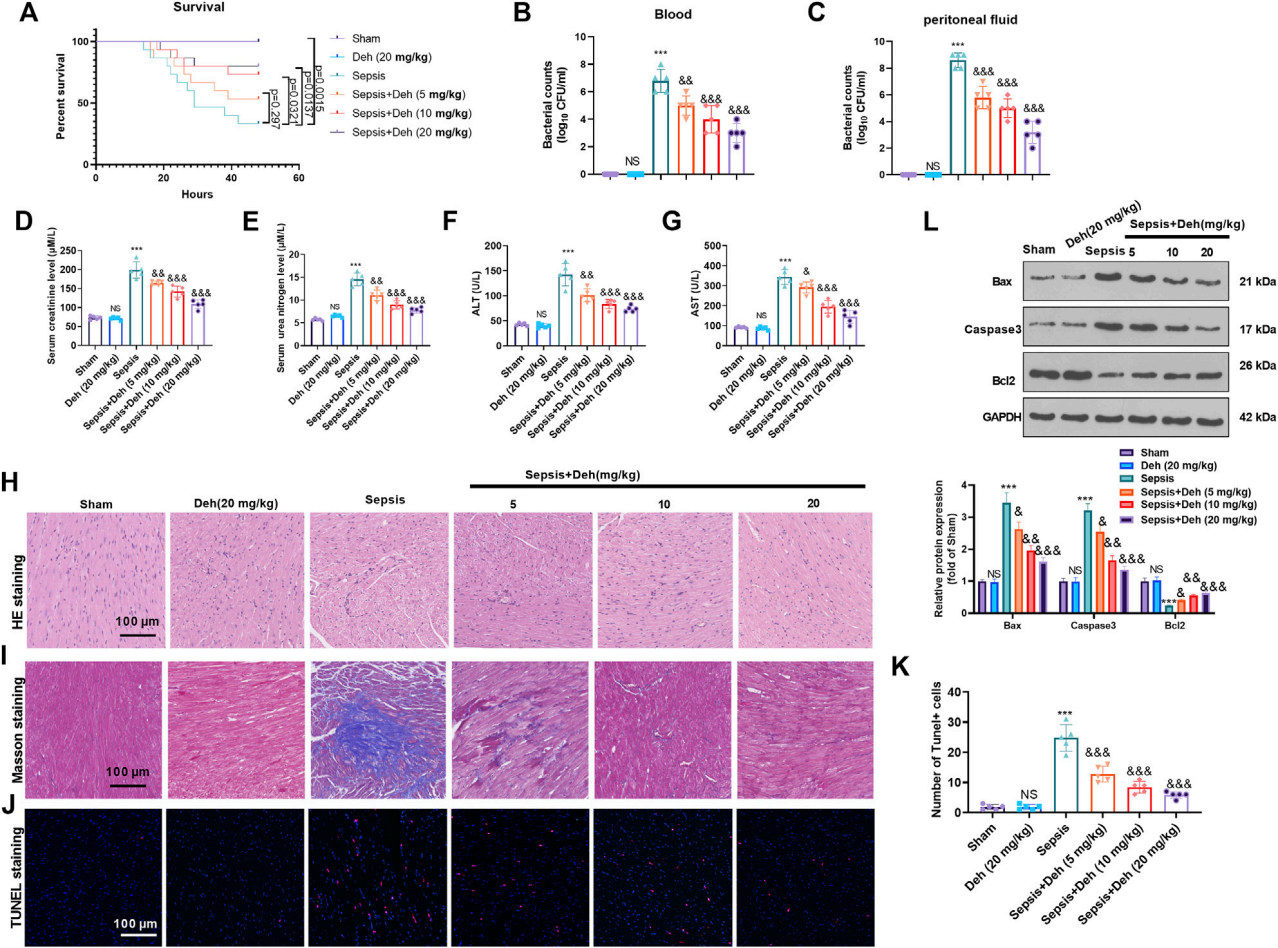
Deh attenuated sepsis-mediated myocardial damage. The *in-vivo* model of sepsis in C57BL/6 mice was induced by intraperitoneal injection of *Escherichia coli* (*E. coli*). Deh (5–20 mg/kg body weight) was administered into the mice *via* intraperitoneal injection. **(A)** The survival (within 48 h after modeling) of mice was analyzed *via* the Kaplan Meier method. **(B, C)** The bacterial counts in the collected peritoneal lavage fluid and serum were calculated. **(D–G)**. The levels of creatinine, urea nitrogen, aspartate aminotransferase (AST), and alanine aminotransferase (ALT) in the supernatant of peritoneal lavage fluid and serum were measured using an automatic biochemical analyzer. **(H, I)** HE staining and Masson staining were used to detect myocardial injury in different groups of mice. **(J, K)** TUNEL-labeled apoptosis cells were detected using the TUNEL-detection kit. The number of TUNEL-positive cell were counted. **(L)** Western blot was carried out to examine the protein levels of Bax, Caspase3 and Bcl2 in the myocardial tissues. NS *p* > 0.05, ***p < 0.001 (vs. Sham group); & *p* < 0.05, && *p* < 0.01, &&&*p* < 0.001 (vs. Sepsis group). N = 5.

To study the effect of Deh on sepsis-induced myocardial injury, E. coli-induced C57BL/6 mice were treated with Deh at different concentrations (5, 10 and 20 mg/kg) for 2 days. First, the survival of mice was analyzed. It was found that the survival rate was significantly reduced compared with the sham group (*p* = 0.0015). With the treatment of Deh, the survival rate increased, and the data had significance when Deh’s dose was over 10 mg/kg (Figure 1A). The bacterial counts in the collected peritoneal lavage fluid and serum were significantly increased in the sepsis group (*p* < 0.001 vs. Sham group, Figures 1B,C). The treatment of Deh markedly reduced bacterial counts both in he collected peritoneal lavage fluid and serum (*p* < 0.01 vs. Sepsis group, Figures 1B,C). The liver and kidney functions were analyzed. The data showed that the mice in the sepsis group had signficantly increased level of creatinine, urea nitrogen, aspartate aminotransferase (AST), and alanine aminotransferase (ALT) in the serum (*p* < 0.001 vs. Sham group, Figures 1D–G). H&E staining and Masson staining results revealed disordered myocardial cell structure, thickened muscle fibers, increased inflammatory cell infiltration and collagen fibers were detected in sepsis mice. Furthermore, Deh notably reduced LPS-induced myocardial injury in mice, and the higher the Deh concentration was, the higher the efficacy was for injury treatment (Figures 1H,I). The myocardial apoptosis was detected by TUNEL-staining and western blot. The data showed that the TUNEL-positive cells, Caspase3 and Bax protein expressions in the heart tissues of sepsis mice increased, and Bcl2 was significantly downregulated (*p* < 0.05 vs. sham group, Figures 1J–L), whereas Deh treatment markedly decreased TUNEL-labeled cardiomyocytes, inhibited Bax and Caspase3 expression, and promoted Bcl2 level in a dose-dependent manner (*p* < 0.05 vs. sepsis group Figures 1J–L). Therefore, Deh exhibited protective effects on sepsis-induced myocardial injury in mice.

### Deh Attenuates Sepsis-Mediated Inflammation and Oxidative Stress

**FIGURE 2:**
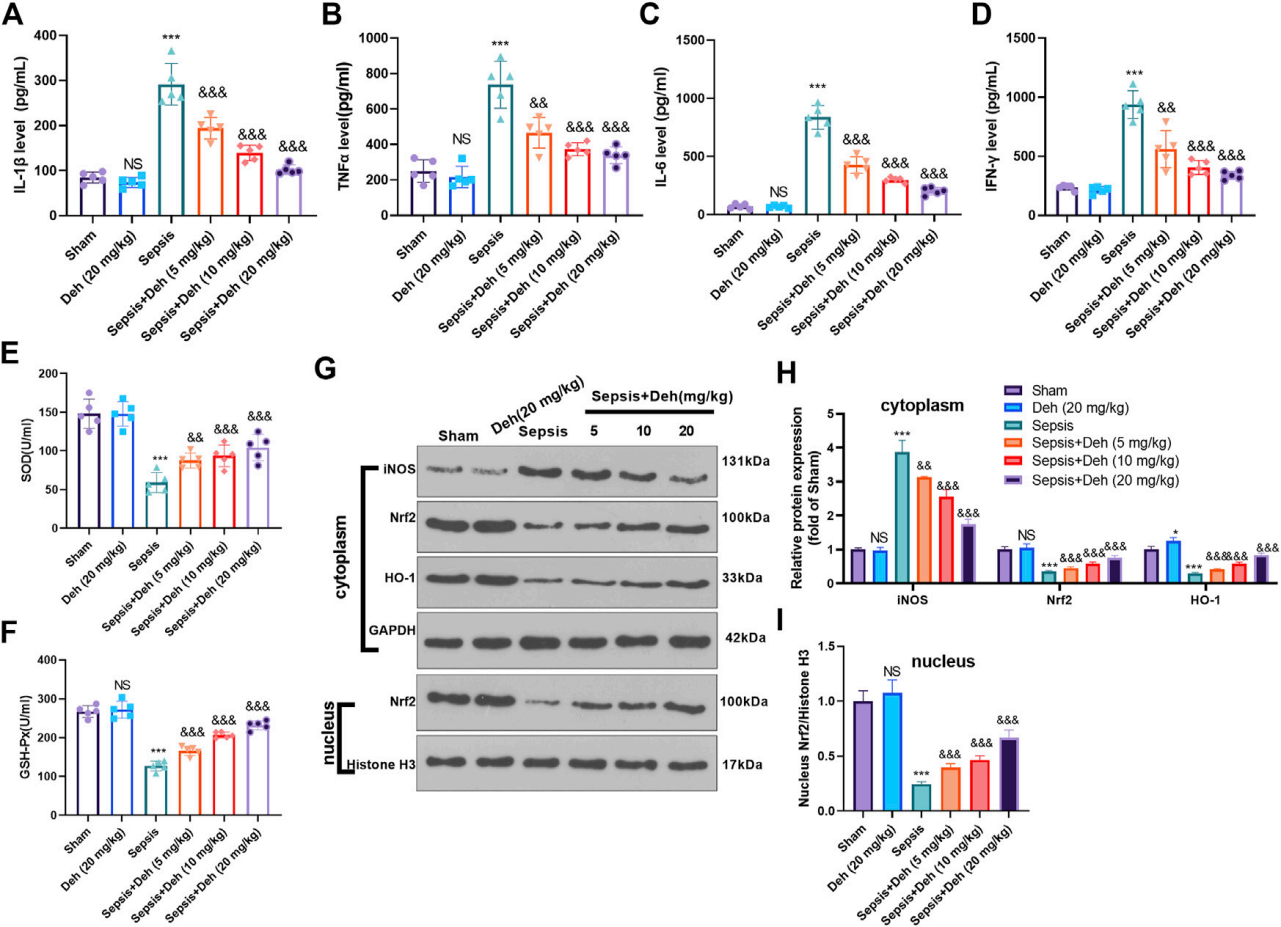
Deh modulated sepsis-mediated inflammation and oxidative stress. **(A–D)** ELISA method was performed to detect IL-1β **(A)**, TNFα **(B)**, IL-6 **(C)**, and IFNγ **(D)** levels in the plasma of mice in each group. **(E, F)** The SOD and GSH-PX detection kits were applied to detect SOD **(E)** and GSH-PX **(F)** levels in the plasma of mice in each group. **(G, I)**. The protein level of iNOS, Nrf2/HO-1 in the whole cell or nucleus of heart was detected by western blot. NS *p* > 0.05, ***p < 0.001 (vs. Sham group); && *p* < 0.01, &&&*p* < 0.001 (vs. Sepsis group). N = 5.

The levels of inflammatory factors (IL-1β, TNFα, IL-6 and IFNγ) and oxidizing factors (SOD and GSH-PX) in the serum were evaluated using the detection kits. The statistical analysis indicated that, compared with those in the sham group, IL-1β, TNFα, IL-6, and IFNγ levels in the Sepsis group were upregulated, while SOD and GSH-PX levels were downregulated (Figures 2A–F). Additionally, Deh treatment reduced IL-1β, TNFα, IL-6, and IFNγ expressions in the plasma in a dose-dependent manner (Figures 2A–D) and increased the expression levels of SOD and GSH-PX (Figures 2E,F). The protein level of Nrf2/HO-1 and iNOS in the heart was detected by western blot. As the data showed, iNOS was significantly increased in the sepsis group, while Nrf2 and HO-1 in the whole cell, and Nrf2 in the nucleus were all repressed (compared with sham group, Figures 2G–I). Interestingly, the administration of Deh significantly repressed iNOS, and promoted cytoplasmic Nrf2, HO-1 and nucleus Nrf2 levels (compared with sepsis group, Figures 2G–I). These results indicated that Deh alleviated the LPS-induced inflammatory response and promoted the expression of antioxidant stress factors.

### Deh Attenuates LPS-Mediated H9C2 Cell Damage

**FIGURE 3:**
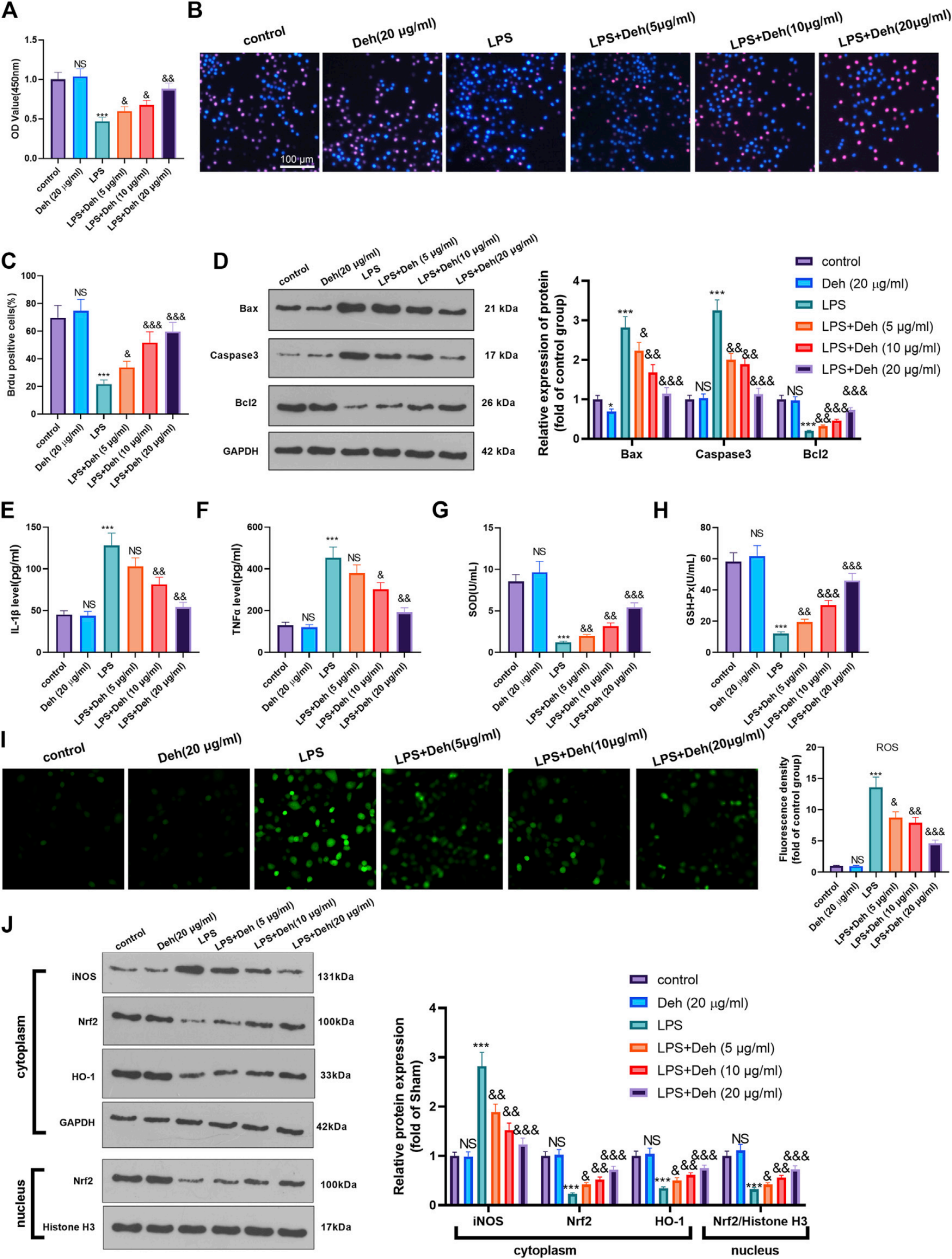
Deh attenuated LPS-mediated H9C2 cell damage. The H9C2 cardiomyocytes were treated with LPS (10 μg/ml) and/or Deh (5–20 μg/ml) for 24 h **(A, B)** CCK8, and BrdU assay were taken to detect the cell viability of each group. **(C)** The rate of BrdU positive cells (red)/nucleus (blue) was calculated. **(D)** Western blot was performed for the detection of Bax, Caspase3 and Bcl2 expressions in cardiomyocytes. **(E, F)** ELISA method was employed to evaluate IL-1β and TNFα levels in the culture medium of each group. **(G, H)** The SOD and GSH-PX detection kits were applied to detect SOD **(G)** and GSH-PX **(H)** levels in the culture medium of each group. I. The ROS level in H9C2 cells were evaluated using the DCFDA/H2DCFDA—Cellular ROS Assay Kit. J. The protein level of iNOS, Nrf2/HO-1 in the whole cell or nucleus of heart was detected by western blot. NS *p* > 0.05, *p < 0.05, ***p < 0.001 (vs. control group); & *p* < 0.05, && *p* < 0.01, &&&*p* < 0.001 (vs. LPS group). N = 3.

An *in-vitro* model of sepsis-induced myocardial injury model was constructed using LPS-induced H9C2 cardiomyocytes. The model was treated with different concentrations of Deh to further investigate the effects of Deh on sepsis-mediated myocardial injury. CCK8 and BrdU assays were carried out to detect the viability of cells in different groups. The results demonstrated that, compared with that in the control group, the cell viability in the Deh group didn’t significantly alter, while in LPS group was considerably reduced. Deh enhanced the viability in a dose-dependent manner compared with that in the LPS group (Figures 3A–C). Subsequently, western blot was performed to investigate the effects of Deh on the expression levels of apoptosis-related proteins, such as Bax, Caspase3 and Bcl2. The results indicated that with the increase in Deh concentration, the expression levels of the pro-apoptotic proteins Bax and Caspase3 were downregulated, whereas the expression levels of the anti-apoptotic protein Bcl2 were upregulated (Figure 3D). Furthermore, the levels of inflammatory factors (IL-1β and TNFα) and antioxidant factors (SOD and GSH-PX) were measured *via* ELISA. The results revealed that Deh reduced IL-1β and TNFα levels in LPS-induced H9C2 cells in a dose-dependent manner, and increased SOD and GSH-PX levels (Figures 3E–H). The ROS level in H9C2 cells was detected. The result indicated that LPS remarkedly promoted ROS level, and Deh attenuated ROS generation in a dose-dependently (Figure 3I). Western blot was conducted to evaluate the protein level of Nrf2/HO-1 and iNOS in the H9C2 cells. It was found that iNOS was significantly increased in the LPS group, while Nrf2 and HO-1 in the whole cell, and Nrf2 in the nucleus were all repressed (compared with control group, Figure 3J). Interestingly, the Deh administration significantly repressed iNOS, and promoted Nrf2, HO-1 and nucleus Nrf2 levels (compared with LPS group, Figure 3J). The aforementioned results indicated that Deh attenuated LPS-mediated H9C2 cell damage *via* repressing inflammation and oxidative stress in a dose-dependent manner.

### Deh Attenuates Activation of the TRAF6/NF-κB Signaling Pathway

**FIGURE 4:**
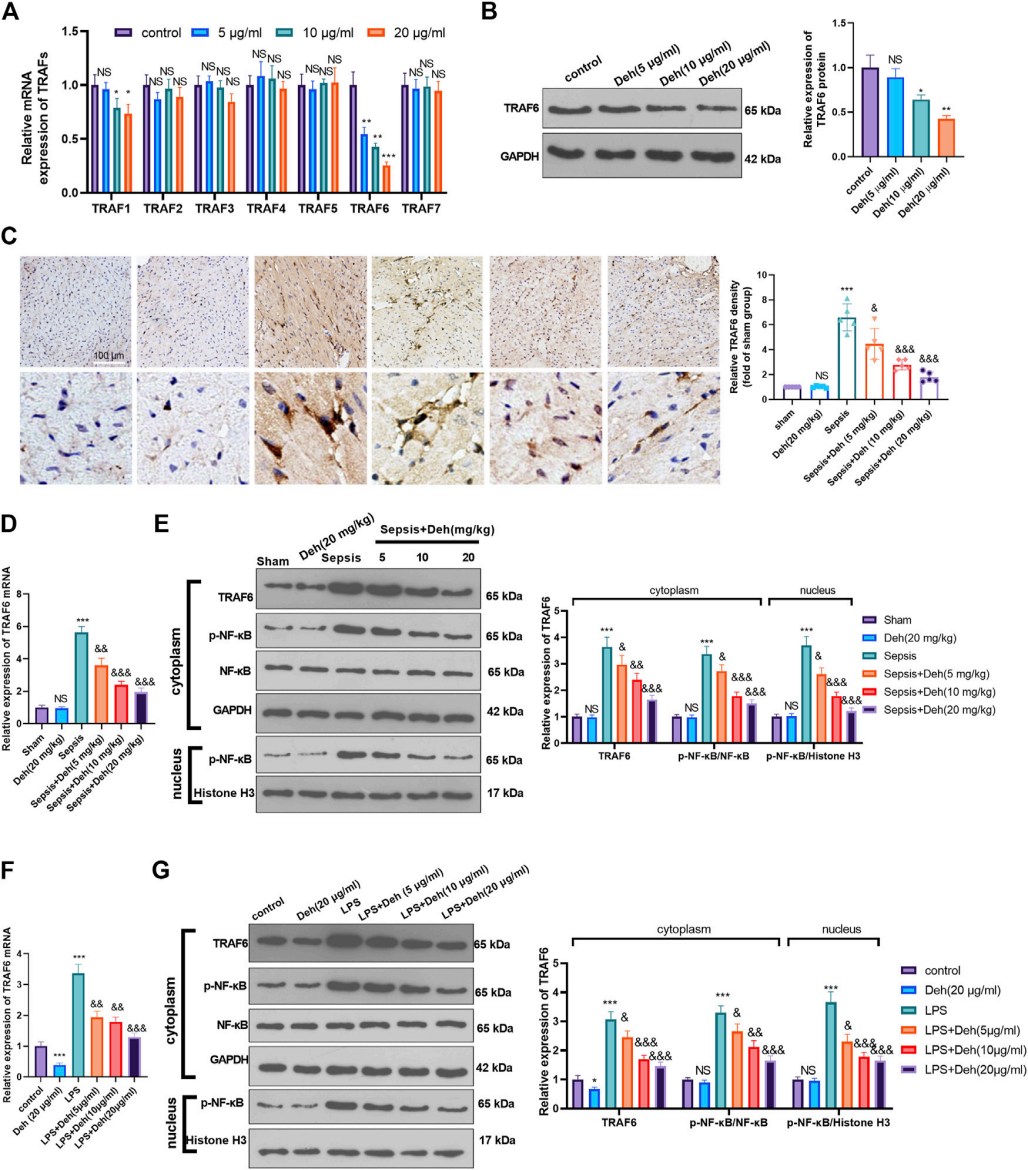
Deh attenuated TRAF6/NF-κB expression. **(A)** RT-PCR was used for evaluating TRAF1∼7 in H9C2 cells treated with Deh (5–20 μg/ml) for 24 h. **(B)** Western blot was performed to examine TRAF6 in H9C2 cells. **(C)** Immunohistochemistry was used to evaluate the expression of TRAF6 in the mouse myocardial tissues of each group, N = 5. **(D, E)** RT-PCR and Western blot was used for evaluating TRAF6 and p-NF-κB expressions in the myocardial tissues of each group, N = 5. **(F, G)** RT-PCR and Western blot was used for evaluating TRAF6 and p-NF-κB expressions in H9C2 cardiomyocytes, N = 3. NS *p* > 0.05, *p < 0.05, **p < 0.01, ***p < 0.001 (vs. control or sham group); & *p* < 0.05, && *p* < 0.01, &&& *p* < 0.001 (vs. LPS group).

The mechanism by which Deh attenuated sepsis-induced myocardial injury was investigated further. First, RT-PCR was used to detect TRAFs (including TRAF1, TRAF2, TRAF3, TRAF4, TRAF5, TRAF6, and TRAF7) in H9C2 cells treated with Deh (5–20 μg/ml). It was found that it was TRAF6, not the other TRAFs, were significantly inhibited with Deh treatment (Figure 4A). The result of western blot also indicated that Deh attenuated TRAF6 protein level in H9C2 cells (Figure 4B). Immunohistochemistry was performed to examine the level of TRAF6 in myocardial tissues. It was found that sepsis induced significant promotion of TRAF6 in the heart, and Deh mitigated TRAF6 upregulation in sepsis mice (Figure 4C). Subsequently, the relative levels of TRAF6 and p-NF-κB in myocardial tissues and H9C2 cells were evaluated using RT-PCR or western blot. It was revealed that TRAF6, p-NF-κB (in the whole cell) and nucleus p-NF-κB were upregulated in both sepsis mouse myocardial tissues and LPS-induced H9C2 cells. Deh treatment decreased TRAF6 and p-NF-κB levels in a dose-dependent manner (Figures 4D–G). These results suggest that Deh attenuates TRAF6/NF-κB signaling pathway activation in sepsis myocardial injury model.

### Inhibition of TRAF6 Attenuates Deh-Mediated Myocardial Protection Against LPS

**FIGURE 5:**
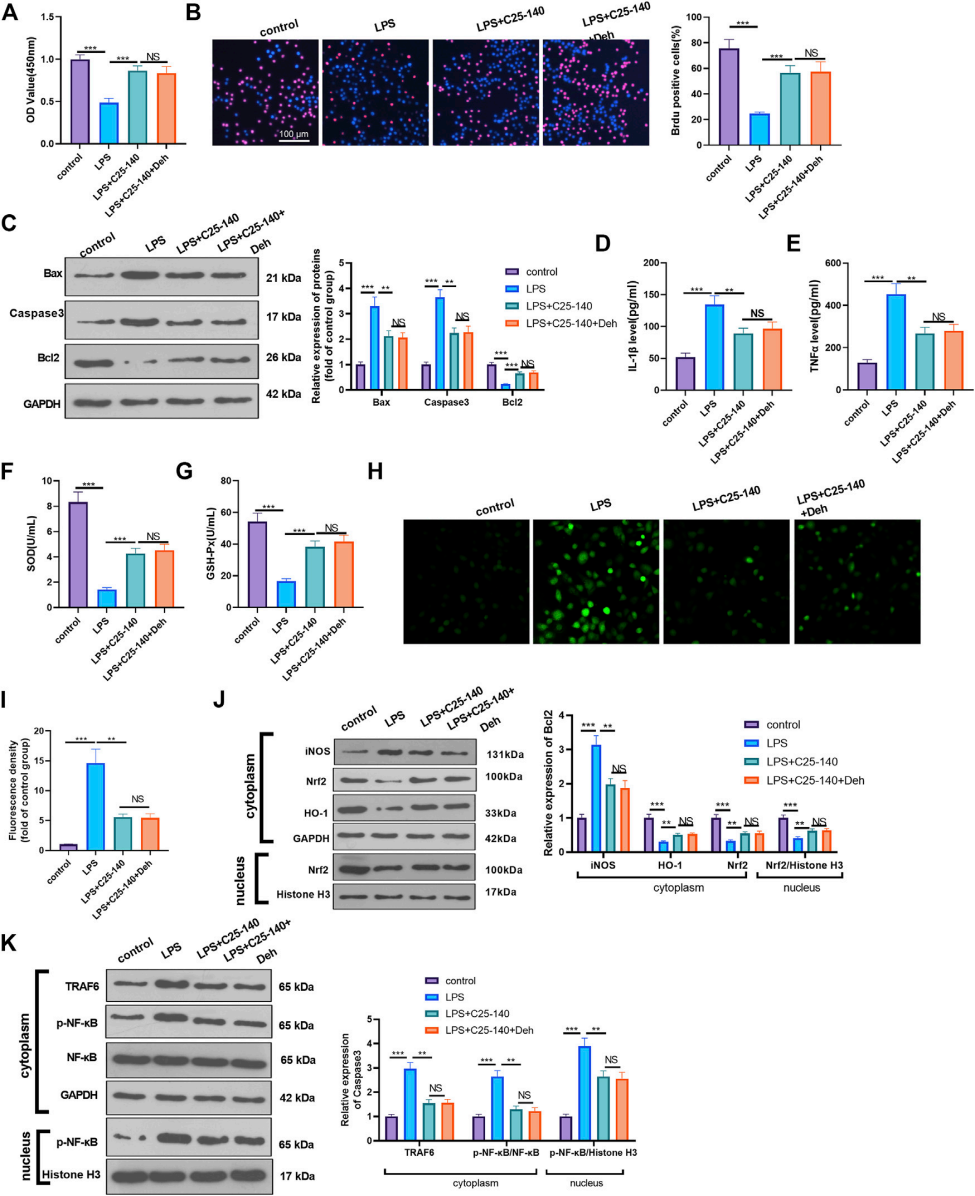
Inhibition of TRAF6 attenuated LPS-mediated myocardial injury. The H9C2 cardiomyocytes were pretreated with C25-140 (5 μM, an inhibitor of TRAF6) for 2 h and then treated with LPS (10 μg/ml) and Deh (10 μg/ml) for 24 h **(A, B)** CCK8, and BrdU assay were taken to detect the cell viability of each group. The rate of BrdU positive cells (red)/nucleus (blue) was calculated. **(C)** Western blot was performed for the detection of Bax, Caspase3 and Bcl2 expressions in cardiomyocytes. **(D, E)** ELISA method was employed to evaluate IL-1β and TNFα levels in the culture medium of each group. **(F, G)** The SOD and GSH-PX detection kits were applied to detect SOD **(G)** and GSH-PX **(H)** levels in the culture medium of each group. **(H, I)**. The ROS level in H9C2 cells were evaluated using the DCFDA/H2DCFDA - Cellular ROS Assay Kit. **(J, K)**. The protein level of iNOS, Nrf2/HO-1, TRAF6, and p-NF-κB in the whole cell or nucleus of heart was detected by western blot. nsP> 0.05, **p < 0.01, ***p < 0.001. N = 3.

To examine whether Deh prevents sepsis-mediated myocardial injury *via* the TRAF6/NF-κB signaling pathway, LPS-induced H9C2 cardiomyocytes were treated with TRAF6 inhibitor C25-140. CCK8 and BrdU assays were performed to detect cell proliferation in different groups, and it was revealed that, compared with the LPS group, the cell viability was enhanced following C25-140 intervention, while no significant difference was observed in the LPS + C25-140 + Deh group compared with the LPS + C25-140 group (Figures 5A,B). Additionally, C25-140 inhibited Bax and Caspase3 expression and decreased Bcl2 expression in LPS-induced cells, while no significant changes were observed with the supplement of Deh in LPS + C25-140-treated cells (Figure 5C). Furthermore, C25-140 inhibited IL-1β, TNFα, and ROS expression and enhanced SOD and GSH-PX expression in LPS-induced cells. However, the cells treated with the combination of C25-140 and Deh exhibited no significant difference in the expression levels of the inflammatory factors and oxidative factors compared with cells in the LPS + C25-140 group (Figures 5D–I). Western blot was applied to detect the levels of iNOS, Nrf2, HO-1, TRAF6 and p-NF-κB in LPS-induced H9C2 cardiomyocytes. It was revealed that C25-140 limited iNOS, TRAF6 and p-NF-κB levels, while promoted Nrf2 and HO-1 expression. However, no significant difference was observed in the relative levels of the above proteins in LPS-induced H9C2 cardiocytes after the addition of Deh (*p* > 0.05, vs. LPS + C25-140 group, Figures 5J,K). The aforementioned results suggest that inhibition of TRAF6 attenuates Deh-mediated myocardial protective effects.

### Inhibition of NF-κB Attenuates Deh-Mediated Myocardial Protection Against LPS

**FIGURE 6:**
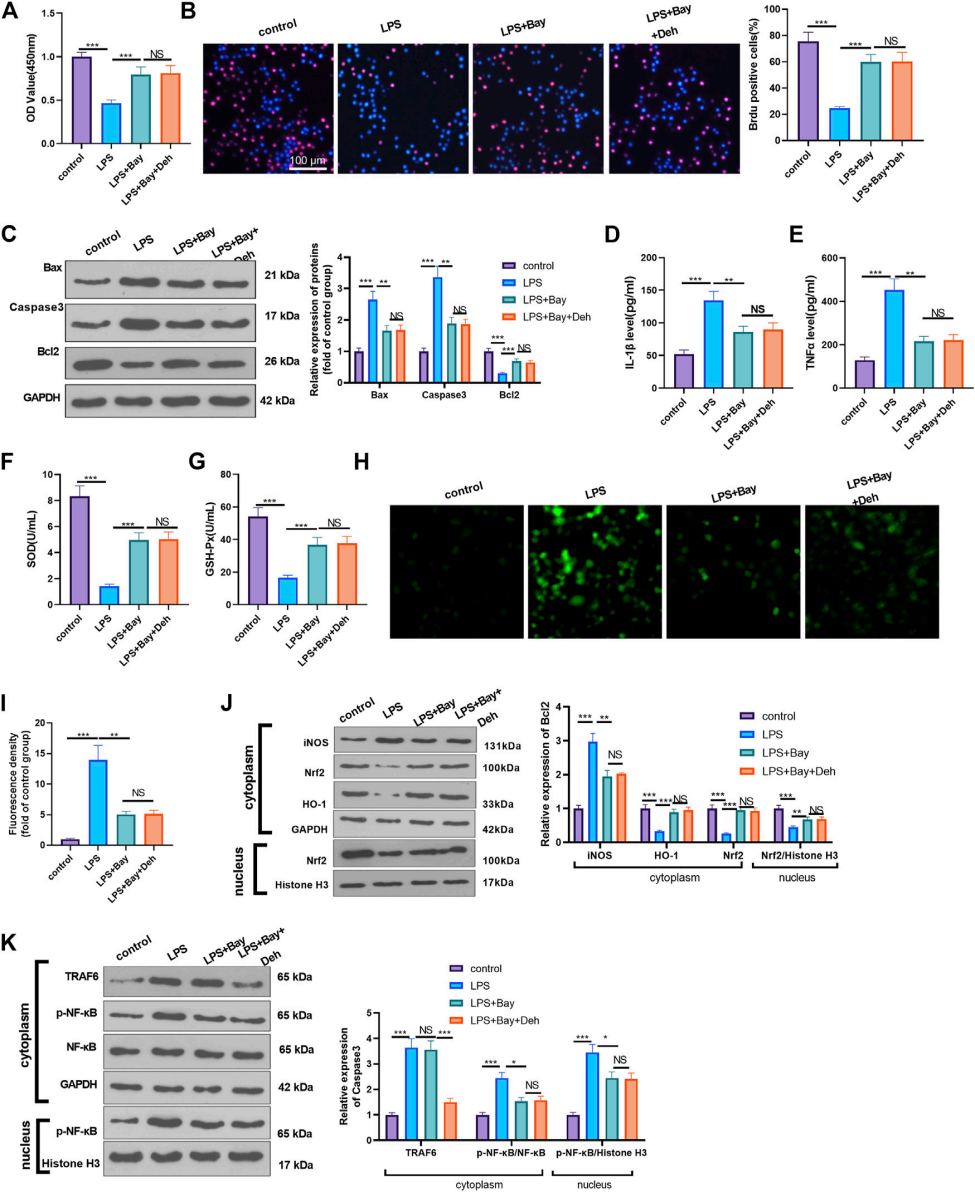
Inhibition of NF-κB attenuated LPS-mediated myocardial injury. The H9C2 cardiomyocytes were pretreated with BAY 11-7082 (BAY, 1 μM, an inhibitor of NF-κB) for 2 h and then treated with LPS (10 μg/ml) and Deh (10 μg/ml) for 24 h **(A, B)** CCK8, and BrdU assay were taken to detect the cell viability of each group. The rate of BrdU positive cells (red)/nucleus (blue) was calculated. **(C)** Western blot was performed for the detection of Bax, Caspase3 and Bcl2 expressions in cardiomyocytes. **(D, E)** ELISA method was employed to evaluate IL-1β and TNFα levels in the culture medium of each group. **(F, G)** The SOD and GSH-PX detection kits were applied to detect SOD **(G)** and GSH-PX **(H)** levels in the culture medium of each group. H-I. The ROS level in H9C2 cells were evaluated using the DCFDA/H2DCFDA—Cellular ROS Assay Kit. **(J, K)**. The protein level of iNOS, Nrf2/HO-1, TRAF6, and p-NF-κB in the whole cell or nucleus of heart was detected by western blot. nsP> 0.05, *p < 0.05, **p < 0.01, ***p < 0.001. N = 3.

Furtermore, LPS-induced H9C2 cells were dealt with the NF-κB inhibitor BAY 11-7082. The proliferation of H9C2 cells was determined by CCK8 and BrdU assays. It was found that BAY11-7082 relieved LPS-mediated cell proliferation decline. However, the combined treatment of BAY 11-7082 and Deh exerted no additional effects on H9C2 cell proliferation (*p* > 0.05 vs. LPS + BAY 11-7082 group, Figures 6A,B). The detection of apoptosis-related proteins revealed that BAY 11-7082 inhibited Bax and Caspase3 expression in LPS-treated H9C2 cells, Bcl2 expression in LPS-induced cells was promoted. The addition of Deh in LPS + BAY 11-7082 group had no significant changes on those proteins (Figure 6C). The inflammatory cytokines, including IL-1β and TNFα, and ROS expression were all repressed by BAY 11-7082 (vs. LPS group), and BAY 11-7082 also enhanced SOD and GSH-PX expression in LPS-induced cells. However, the cells treated with the combination of BAY 11-7082 and Deh exhibited no significant difference in the expression levels of the inflammatory factors and oxidative factors compared with cells in the LPS + BAY 11-7082 group (Figures 6D–I). The levels of iNOS, Nrf2, HO-1, TRAF6 and p-NF-κB in LPS-induced H9C2 cardiomyocytes were evaluated by Western blot. It was shown by the results that BAY 11-7082 suppressed iNOS, and p-NF-κB levels, while promoted Nrf2 and HO-1 expression. However, no significant difference was observed in the relative levels of the above proteins in LPS-induced H9C2 cells after the addition of Deh (*p* > 0.05, vs. LPS + BAY 11-7082 group, Figures 6J,K). Collectively, those data suggested that Deh relieved LPS-mediated inflammatory, oxidative and H9C2 cell apoptosis dependently through inhibiting NF-κB pathway.

## Discussion

The present study explored the therapeutic effects of Deh in an LPS-induced sepsis model. The present data indicated that Deh ameliorated myocardial injury by modulating inflammation and oxidative stress through repressing TRAF6-mediated NF-κB pathway.

Sepsis is a systemic inflammatory response syndrome, and is caused by severe infections of various sources. Multi-organ functional injury is a major complication of sepsis, among which myocardial injury and cardiac failure are the most common complications of sepsis and are also one of the major risk factors affecting the prognosis of severe sepsis (Frencken et al., 2018). Currently, research focuses on how to minimize myocardial injury in severe sepsis and protect cardiac function (Kakihana et al., 2016, Ito, Nakahara, Yamaguchi, Yasuda; Fernandes et al., 1999). With the increased researches on myocardial injury, multiple drugs have been found effective against it *via* repressing inflammation, and oxidative stress. For example, salidroside can exert a protective effect on LPS-induced myocardial injury by inhibiting the reactive oxygen species-mediated PI3K/AKT/mTOR signaling pathway (Chen et al., 2017). Furthermore, oxymatrine inhibits the Janus kinase 2/STAT3 signaling pathway and decreases the expression levels of pro-inflammatory factors IL-1β and TNFα, thereby alleviating myocardial injury caused by septic shock (Zhang et al., 2013). Delta opioid receptor agonists can alleviate LPS-induced myocardial ultrastructure damage in mice, protect against myocardial insufficiency and improve the survival rate of mice (Zhao et al., 2017). Moreover, many traditional Chinese medicines have been found with anti-inflammatory and antioxidant effects (Shingnaisui et al., 2018). Hence, it is of great significance to investigate the protective role of traditional Chinese medicine extracts in sepsis-mediated myocardial injury.

Deh is an important bioactive component of the Chinese herb *Corydalis ambigua*. This herb has a wide range of therapeutic effects in a variety of diseases, including cancer associated pain (Huo et al., 2018), depression (Jin et al., 2019), melanoma (Hu et al., 2019), and so on. For example, Deh stimulates MAPK activation of p38 protein and enhances the interaction between MyoD and E protein, thus leading to the activation of MyoD and myoblast differentiation, and enhancing the regeneration ability of injured muscle stem cells (Yoo et al., 2016). Yin et al Yin et al. (2016) reported that Deh could reduce the formalin-induced pain response in mice and decreased the expression levels of IL-1β, IL-6 and other inflammatory factors. Ishiguro et al Ishiguro et al. (2011) revealed that Deh not only inhibited the increase of mitochondrial membrane potential and ATP depletion in LPS-stimulated macrophages, but also inhibited the increase in IL-1β and IL-6 concentration in LPS-induced macrophage culture medium. The present study revealed that Deh enhanced viability and inhibited apoptosis of cardiomyocytes induced by LPS *in vitro* or *Escherichia coli* in mice. Furthermore, Deh attenuated IL-1β and TNFα levels in plasma and cardiomyocyte culture medium in a dose-dependent manner and increased the expression levels of SOD and GSH-PX. These results suggest that Deh attenuates sepsis-mediated myocardial injury *via* anti-inflammation and anti-oxidative stress.

TNF receptor-associated factor family members mainly include TRAF1-7. Interestingly, previous studies have found that the TRAFs, including TRAF1 (Bin et al., 2019), TRAF2 (Etemadi et al., 2015), TRAF3 (Lei et al., 2021), TRAF4 (Li et al., 2020), TRAF5 (Sun et al., 2021), TRAF6 (Min et al., 2018) and TRAF7 (Zotti et al., 2011) all have a role in modulating NF-κB activity. The upregulation of TRAFs also makes a role during sepsis progression. For example, LPS and TNFalpha increased nuclear translocation of NF-κB p65 dependently throough enhancing the steady state of TRAF1 mRNA in neutrophil (PMN) (Nolan et al., 2000). In another study, inflammatory preconditioning (InP) induces TLR9 translocation from the neutrophil cytosol to the membrane. Thus, TLR9 binds to Cav-1 and activates MyD88-mediated TRAF3 and IRF3 signal transduction (Yang et al., 2019). Presently, we detected all of the seven TRAFs in H9C2 cells treated by different doses of Deh. It was found that TRAF6 was significantly downregulated by Deh. Interestingly, Previous studies (Wullaert et al., 2007; Chen et al., 2010) have reported that LPS, a ligand of TLR4, activates the complex of IL-1β receptor-associated kinases and TRAF6 after binding with it, and TRAF6 releases TNFα, IL-6 and other pro-inflammatory factors by inducing the nucleus translocation of phosphorylated NF-κB p65 (Dai et al., 2017). It has been found that blocking TRAF6 limits the inflammatory response-mediated by intracranial hemorrhage (Yang et al., 2020), Alzheimer’s disease (Wang et al., 2018), and *Salmonella typhimurium* infection (Min et al., 2017). Furthermore, we explored the underlying mechanism of TAF6/NF-κB pathway in sepsis-induced myocardial damage. The present study revealed that sepsis promoted TRAF6 expression and the nucleus translocation of phosphorylated NF-κB p65. Deh treatment decreased the levels of TRAF6 and p-NF-κB p65 in a dose-dependent manner. Both of the TRAF6 inhibitor of C25-140 and NF-κB inhibitor BAY 11-7082 relieved LPS-mediated injury on H9C2 cells, while the addition of Deh gained no more protective effects. Those data suggests that Deh has a protective effect on LPS-induced myocardial cell injury dependently through inhibiting the TRAF6/NF-κB signaling pathway.

Oxidative stress plays a vital role in sepsis-induced organ injury (Nadeem et al., 2021). Those overproduced reactive species and/or free radicals, including nitric oxide (NO), peroxynitrite (ONOO^−^), superoxide (O_2_^−^), hydrogen peroxide (H_2_O_2_), and hydroxyl radical (OH), are the results of impairment of oxygen utilization by cells and limited delivery of oxygen to tissues (Prauchner, 2017). Nuclear factor erythroid 2-related factor 2 (Nrf2), a member of the Cap-n-Collar family of basic leucine zipper proteins, is crucial in antioxidant defense *via* controlling the basal and induced expression of an array of antioxidant response element-dependent genes [such as heme oxygenase 1 (HO-1)] in both physiological and pathophysiological environment (Ma, 2013; Mei et al., 2020). In addition, the activation of NF-κB pathway or ROS promotes the expression of inducible nitric oxide synthase (iNOS), which is inhibited by the upregulation of Nrf2/HO-1 pathway (Dijkstra et al., 2004; Shen et al., 2021). Here, we found that sepsis led to enhanced iNOS level and reduced Nrf2/HO-1 expression in cardiomyocytes. While all of TRAF6 inhibitor C25-140, NF-κB inhibitor BAY 11-7082 and Deh treatments reduced iNOS level and enhanced Nrf2 expression (both in the whole cell and nucleus). Therefore, we believed that Deh has anti-oxidative stress effects *via* activating Nrf2/HO-1 pathway.

**FIGURE 7:**
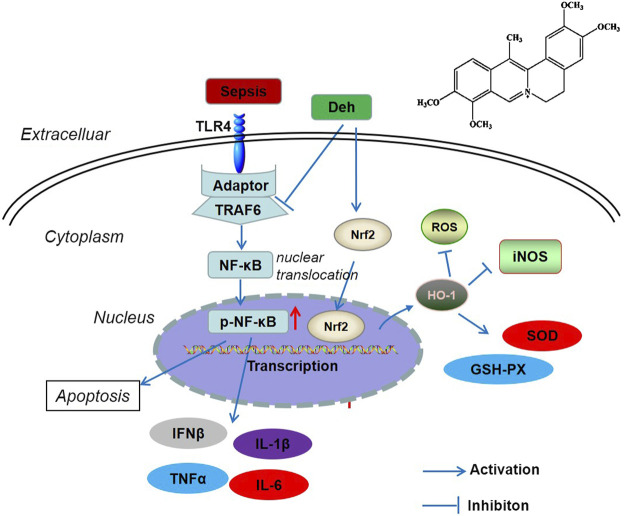
The scheme of Deh mediated myocardial protective effects against sepsis. LPS was recognized by TLR4 and then activated TRAF6 mediated NF-κB phosphorylation. The phosphorylated NF-κB translocated from the cytoplasm into the nucleus and promoted the transcription of inflammatory factors. Once Deh was administered, TRAF6 was in inhibited and the NF-κB mediated inflammation was also attenuated. Moreover, Deh treatment promoted Nrf2 nucleus translocation. Nrf2 promoted HO-1 expression, which induced enhanced anti-oxidative meidators (including SOD, GSH-PX) and inhibited iNOS and ROS level.

In conclusion, the present study revealed that Deh attenuated sepsis-mediated myocardial injury. By inhibiting the activation of the TRAF6/NF-κB signaling pathway and activating Nrf2/HO-1 pathway, Deh reduced the release of pro-inflammatory factors and increased the levels of antioxidant stress factors (Figure 7). The present study provides novel insights for the treatment of sepsis-mediated myocardial injury. However, further experiments are required to determine the mechanisms of Deh-mediated myocardial protective effects.

## Data Availability

The raw data supporting the conclusions of this article will be made available by the authors, without undue reservation.
